# Respiratory Physiotherapy in Preterm Neonates with Bronchopulmonary Dysplasia or Respiratory Distress Syndrome: A Comprehensive Review of Clinical Evidence and Therapeutic Implications

**DOI:** 10.3390/jcm15010343

**Published:** 2026-01-02

**Authors:** Paula Rodríguez-Roza, Raquel Leirós-Rodríguez, Arrate Pinto-Carral, María José Álvarez-Álvarez

**Affiliations:** 1 Department of Rehabilitation, Hospital Centro Médico de Asturias, 33193 Oviedo, Principado de Asturias, Spain; paularodriguezroza@hotmail.com; 2 SALBIS Research Group, Department of Nursing and Physiotherapy, Universidad de León, 24401 Ponferrada, León, Spain; rleir@unileon.es

**Keywords:** preterm neonates, respiratory disorders, physiotherapy

## Abstract

**Background:** Preterm birth, affecting more than 13.4 million infants worldwide each year, remains one of the leading causes of neonatal morbidity and mortality. Among its complications, respiratory distress syndrome and bronchopulmonary dysplasia are predominant contributors to prolonged hospitalization and respiratory support needs. As advances in perinatal care have improved survival, attention has increasingly turned to optimizing respiratory function and reducing complications through non-pharmacological interventions. Respiratory physiotherapy has therefore gained recognition as a valuable adjunct to medical management in this population. **Purpose:** To provide a comprehensive synthesis of the current clinical evidence regarding respiratory physiotherapy techniques used in preterm neonates with respiratory distress syndrome or bronchopulmonary dysplasia. **Summary of Evidence:** The available literature describes several physiotherapeutic modalities—including prolonged slow expiration, postural treatment, Vöjta therapy, and gentle mechanical techniques—aimed at improving ventilation, gas exchange, and secretion clearance. Across diverse studies, these interventions have been associated with better oxygenation, improved heart and respiratory rates, shorter mechanical ventilation time, and reduced hospital stay, while showing no relevant adverse effects. Although methodological heterogeneity persists, the consistency of beneficial trends supports their integration into multidisciplinary neonatal care. **Conclusions:** Respiratory physiotherapy represents a safe and promising therapeutic complement for preterm neonates with respiratory distress syndrome or bronchopulmonary dysplasia. Techniques that combine postural control and controlled expiratory maneuvers appear particularly effective in enhancing pulmonary mechanics and recovery. Future research should focus on standardizing intervention protocols, identifying optimal timing and dosing, and evaluating the long-term respiratory and developmental outcomes of these physiotherapeutic strategies.

## 1. Introduction

Preterm birth remains a major global health challenge. In 2020, an estimated 13.4 million babies, approximately 1 in 10 births worldwide, occurred before 37 weeks’ gestation, with little improvement in rates over the last decade [1].

Prematurity is the leading cause of neonatal mortality and contributes substantially to morbidity, prolonged hospitalization, and long-term developmental impairment [2]. In 2022, global neonatal mortality reached 2.3 million deaths, nearly half of all deaths among children under five [2].

Epidemiological analyses consistently show that the burden of prematurity is high across countries and healthcare systems. However, prevalence and risk profiles vary depending on population and healthcare context. The epidemiological burden of prematurity is not evenly distributed worldwide.

Rates of preterm birth and associated neonatal mortality are substantially higher in low- and middle-income countries, where access to prenatal care, neonatal intensive care, and advanced respiratory support may be limited, whereas high-income countries report lower mortality but a higher prevalence of long-term morbidity among survivors.

A recent study reported a 23% prevalence of prematurity among all live births admitted to a reference neonatal intensive care unit (NICU), reflecting a hospital-based population rather than the general newborn population [3].

These findings reflect a hospital-based population rather than the general newborn population and highlight the multifactorial nature of prematurity, with primigravidity and hypertensive disorders of pregnancy identified as major risk factors, while adequate prenatal care appears to act as a protective factor [2,3].

Among the clinical complications associated with prematurity, respiratory morbidity is particularly prevalent and clinically relevant. Neonatal respiratory distress syndrome (RDS) remains one of the most frequent causes of acute respiratory failure in preterm infants, with incidence rates reaching 40–70% in very low birth-weight neonates, depending on gestational age [4].

In contrast, bronchopulmonary dysplasia (BPD) represents the main chronic pulmonary sequela of prematurity, emerging from the interaction of lung immaturity, oxygen exposure, infection, and ventilator-induced lung injury, and is typically considered a chronic condition rather than an acute respiratory disorder. It is frequently associated with long-term respiratory impairment and increased healthcare utilization [5].

Despite advances in antenatal corticosteroids, surfactant therapy, non-invasive ventilation and lung-protective strategies, the prevalence of both RDS-related morbidity and BPD remains substantial [6]. Consequently, current neonatal care increasingly emphasizes not only survival but also the prevention of lung injury, reduction in invasive ventilation, and optimization of long-term pulmonary development.

Taken together, the high prevalence of acute and chronic respiratory complications in preterm infants, combined with their potential long-term impact on pulmonary function and health-related quality of life, underscores the need for strategies that go beyond short-term survival. Current neonatal care, therefore, increasingly focuses on minimizing lung injury, reducing exposure to invasive ventilation, and promoting optimal long-term respiratory development.

In this context, respiratory physiotherapy has gained attention as a non-pharmacological and non-invasive adjunct therapy in neonatal care. Contemporary approaches focus on gentle, low-pressure techniques aimed at improving ventilation distribution and airway clearance, including prolonged slow expiration, expiratory flow increase, postural management, and selected manual mobilization strategies, such as thoracoabdominal facilitation and reflex rolling, as described in the literature [7,8,9].

It is important to distinguish between traditional chest physiotherapy techniques, such as classical percussion and vibration, and contemporary gentle manual techniques currently applied in neonatal care. Modern standards increasingly discourage aggressive approaches in preterm infants due to their physiological vulnerability and potential risk of adverse effects. Current practice, therefore, emphasizes low-pressure, carefully dosed, and individualized techniques adapted to the infant’s gestational age, clinical stability, and respiratory support.

Previous evidence suggests potential benefits in oxygenation, respiratory and heart rate stabilization, reduction in atelectasis, decreased duration of mechanical ventilation and shorter hospitalization, generally without significant adverse events [7,8,9,10,11].

Nevertheless, the evidence remains heterogeneous. Clinical trials report variable protocols, different treatment frequencies and inconsistent endpoints, limiting the ability to draw firm conclusions. Safety concerns persist, especially in extremely preterm infants, stemming from isolated reports of iatrogenic injuries with inappropriate or vigorous techniques [12].

Reported adverse events in the literature include rib fractures, intracranial hemorrhage, transient hypoxemia, and hemodynamic instability, predominantly described in association with outdated or excessively vigorous techniques, such as forceful percussion or inappropriate chest compression. These events have been linked to improper application, lack of adaptation to gestational age and clinical stability, or insufficient professional training, rather than to contemporary gentle techniques performed according to current neonatal standards [12].

In addition, respiratory physiotherapy is not uniformly applied in all neonatal units. In many centers, its use is based on individual clinical indications rather than routine application, taking into account gestational age, respiratory stability, type of respiratory support, and specific therapeutic goals. This indication-based approach further contributes to optimizing safety and clinical relevance [12,13].

A recent systematic review found that respiratory physiotherapy consistently improved pulmonary mechanics, oxygenation and short-term physiological stability, but again emphasized the lack of standardized methodology and the need for high-quality long-term studies [13].

Marked heterogeneity was observed across studies in terms of intervention protocols, which substantially limits direct comparison of results. Session frequency ranged from one to three daily sessions, while session duration varied from very brief interventions of approximately 3 min to sessions lasting up to 20 min. In addition, the total duration of intervention programs ranged from one week to one month. Considerable variability was also noted in the type and combination of techniques applied, as well as in the clinical context (mechanically ventilated infants, infants on CPAP, or non-ventilated preterm neonates) [13].

Furthermore, the frequent use of small sample sizes across studies limits statistical power and may partly explain inconsistent or non-significant findings, thereby weakening the overall strength of the available evidence.

Respiratory physiotherapy in preterm infants is currently delivered within the context of modern respiratory support strategies, particularly non-invasive modalities such as nasal continuous positive airway pressure and high-flow nasal cannula. As a result, physiotherapy techniques are increasingly applied alongside externally delivered positive end-expiratory pressure [6,7]. This clinical context has raised growing interest in how manual respiratory techniques—especially those aiming to increase expiratory flow—may interact with respiratory support settings, highlighting the need for careful integration and individualized application.

Despite the growing interest and expanding literature, no clear consensus currently exists regarding which respiratory physiotherapy modalities should be routinely implemented, how they should be integrated with modern respiratory support, or which preterm subgroups benefit most. Existing reviews often combine term and preterm infants, focus on only one technique, or pre-date recent randomized trials.

The aim of this comprehensive narrative review is to provide an updated and clinically oriented synthesis of respiratory physiotherapy techniques in preterm neonates with RDS or BPD, focusing on their proposed mechanisms of action, effects on pulmonary function and physiological stability, interaction with respiratory support, and safety profile.

Based on the available evidence, we hypothesize that appropriately indicated and carefully applied respiratory physiotherapy may contribute to short-term improvements in ventilatory parameters and respiratory stability and may influence clinically relevant outcomes such as duration of respiratory support and length of hospitalization, while acknowledging that evidence regarding long-term outcomes, including BPD incidence, remains limited.

## 2. Materials and Methods

### 2.1. Study Design

This study was designed as a narrative review with a structured search strategy. Although the literature search, study selection, and data extraction followed predefined and transparent procedures, the review was not conducted as a formal systematic review. Specifically, no review protocol was registered (e.g., in PROSPERO), no formal risk-of-bias assessment was performed, and no meta-analysis was planned or undertaken. This approach allows for a comprehensive and clinically oriented synthesis of the literature.

### 2.2. Search Strategy

A structured literature search was conducted between November 2024 and January 2025 in the following electronic databases: Web of Science (WOS), PubMed, Scopus, Cochrane Library, PEDro and ScienceDirect.

The search strategy combined Medical Subject Headings (MeSH) and free-text terms related to prematurity, neonatal respiratory disease and respiratory physiotherapy. MeSH terms included *“preterm”*, *“premature”*, *“bronchopulmonary dysplasia”*, *“respiratory distress syndrome”*, *“hyaline membrane disease”*, *“pulmonary diseases”*, *“physical therapy”*, and *“physiotherapy”*. To enhance sensitivity, additional free terms such as *“respiratory diseases”*, *“rehabilitation”*, and *“chest physiotherapy”* were incorporated.

Boolean operators were applied as follows: OR to combine synonyms or related concepts; AND to link population, condition and intervention terms.

As an example, the search strategy used in PubMed was as follows: ((“Preterm Infant”[MeSH] OR preterm OR premature) AND (“Respiratory Physiotherapy” OR “Chest Physiotherapy” OR “Respiratory Therapy”) AND (“Hyaline Membrane Disease”[MeSH] OR “Respiratory Distress Syndrome” OR “Bronchopulmonary Dysplasia”)).

### 2.3. Eligibility Criteria

Articles were selected based on predefined eligibility criteria.

Inclusion criteria:

1. Population: preterm infants (<37 weeks of gestation) with a medical diagnosis of RDS or BPD.
2. Intervention: any respiratory physiotherapy technique or modality.
3. Study design: randomized controlled trials. Only randomized controlled trials were included to ensure a minimum level of internal validity and patient safety, given the clinical vulnerability of preterm neonates and the potential risks associated with respiratory physiotherapy interventions.
4. Publication period: January 2014 to January 2025.
5. Language: English or Spanish, based on feasibility and the predominance of relevant literature.
6. Publication type: Articles published in peer-reviewed journals.

Exclusion criteria:

1. Preterm infants presenting major comorbidities (e.g., congenital malformations, genetic syndromes, significant cardiovascular abnormalities).
2. Studies for which full-text access was not available.
3. Grey literature (theses and conference proceedings).

### 2.4. Study Selection and Data Extraction

Study selection was performed by a single reviewer (P.R.R.), with a second reviewer (M.J.A.A.) consulted whenever uncertainty arose regarding eligibility.

Given the comprehensive and narrative nature of this review, studies were selected based on their relevance, clinical content and contribution to understanding the effects of respiratory physiotherapy on preterm infants with RDS or BPD.

All retrieved records were exported to Mendeley, where duplicate entries were identified and removed. Screening proceeded in two stages: (1) Title and abstract screening, to exclude clearly irrelevant studies or those not meeting basic eligibility criteria, and (2) full-text assessment, to confirm compliance with all inclusion and exclusion criteria.

For each included study, the following information was manually extracted: authorship, year of publication, sample size and characteristics, intervention details, outcome measures, principal findings and authors’ conclusions. Extracted data were compiled into a structured table to support qualitative synthesis and comparison across studies (Table S1).

### 2.5. Quality Assessment

Although this comprehensive review did not include a formal risk-of-bias assessment, methodological considerations and potential sources of bias were critically appraised qualitatively during data interpretation, taking into account study design, reporting clarity and consistency of findings. This approach is consistent with the methodological expectations for comprehensive and narrative reviews, which emphasize breadth of coverage, critical interpretation and integration of evidence rather than quantitative synthesis.

### 2.6. Use of Generative Artificial Intelligence

Generative artificial intelligence (GenAI) tools were used exclusively for the creation of selected graphical elements, including conceptual diagrams and illustrative figures. These tools did not influence study selection, data extraction, methodological decisions, analysis, or interpretation of results. All scientific content was critically reviewed, verified and edited by the authors, who take full responsibility for the final manuscript.

## 3. Manual Techniques for Enhancing Ventilatory Mechanics and Expiratory Flow

Manual respiratory physiotherapy techniques constitute the most extensively represented group of interventions among the studies included in this review. These techniques—applied with gentle, controlled maneuvers—seek to optimize expiratory flow, mobilize secretions, re-expand atelectatic areas and support more efficient ventilatory mechanics in premature infants with respiratory distress.

**Figure 1:**
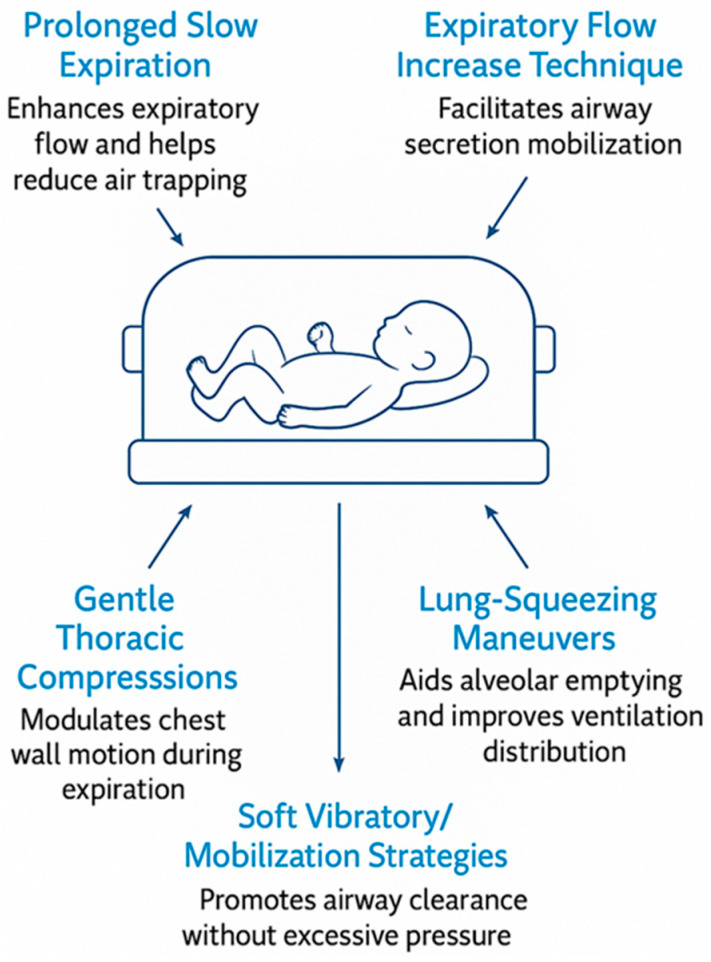
Manual respiratory physiotherapy techniques for preterm infants.

Across the analyzed trials, clinicians employed a variety of manual methods (Figure 1) such as prolonged slow expiration (PLE), the Expiratory Flow Increase Technique (EFIT), gentle thoracic compressions, lung squeezing maneuvers, and soft vibratory or mobilization strategies [14,15,16,17,18,19,20,21]. All of these were delivered in synchrony with spontaneous breathing, respecting the physiological fragility of the premature lung and aiming to modulate expiratory flow without generating excessive intrathoracic pressure.

In a randomized trial involving mechanically ventilated preterm infants, a research group in Brazil demonstrated that EFIT and conventional chest physiotherapy produced immediate improvements in oxygen saturation (SpO_2_), decreased respiratory rate and reduced pain scores following treatment [14]. Further physiological benefits were reported in a study led by an Indian neonatal team, where a single session of chest mobilization improved ventilatory patterns and reduced the work of breathing both in extubated and ventilated infants [15].

Manual techniques were also applied with the goal of resolving atelectasis, a frequent complication in RDS. In a clinical investigation focused on lobar collapse, lung squeezing contributed to significant radiological improvement and better oxygenation profiles within 24 h of treatment [20]. Similarly, another trial conducted on extremely preterm infants with severe respiratory distress documented earlier normalization of breath sounds and improved ventilatory patterns when gentle chest physiotherapy techniques were used [17].

It is important to note that all contemporary studies reported excellent safety, with no adverse events observed when techniques were performed by trained neonatal physiotherapists. Historical reports of rib fractures after vigorous compressions [11] highlight the need for expertise, but they do not reflect current professional practice.

Overall, the evidence suggests that well-executed manual techniques can yield rapid and clinically meaningful improvements in oxygenation, thoracoabdominal synchrony, secretion mobilization and general respiratory stability.

## 4. Positioning and Postural Management in Respiratory Support

Positioning strategies represent some of the safest and most physiologically grounded interventions available for premature infants. Because neonatal ribcage structure, diaphragmatic function and lung compliance are highly sensitive to gravity, therapeutic positioning can markedly influence ventilation distribution and gas exchange.

Multiple studies show that specific postures lead to meaningful improvements in respiratory function (Table 1). In a randomized clinical trial conducted in Indonesia, the quarter-prone position significantly increased SpO_2_ in preterm infants receiving continuous positive airway pressure (CPAP), demonstrating more favorable lung recruitment than the supine posture [21]. Comparable benefits were documented in a Brazilian study evaluating CPAP users, where prone and lateral positions promoted better thoracoabdominal synchrony and more homogeneous ventilation distribution [22].

Earlier physiological evidence, such as research carried out in the United States with preterm infants hospitalized for lung disease, also indicated that prone positioning increases lung volumes and reduces areas of dorsal atelectasis compared with the supine position [23]. These findings align with neonatal physiology: prone posture improves diaphragmatic efficiency, reduces anterior ribcage distortion and helps maintain functional residual capacity.

A particularly significant contribution comes from a multicenter clinical trial comparing conventional respiratory physiotherapy to an individualized postural care program. In this investigation, infants receiving tailored positioning strategies experienced a greater reduction in mechanical ventilation time than those assigned to chest physiotherapy, underscoring the therapeutic value of optimized posture even in critically ill infants [19].

Positioning, therefore, emerges as a foundational respiratory support tool, non-invasive, physiologically sound and applicable to virtually all preterm infants, particularly those in whom excessive handling must be avoided.

## 5. Reflex-Based and Neurodevelopmental Approaches

**Figure 2:**
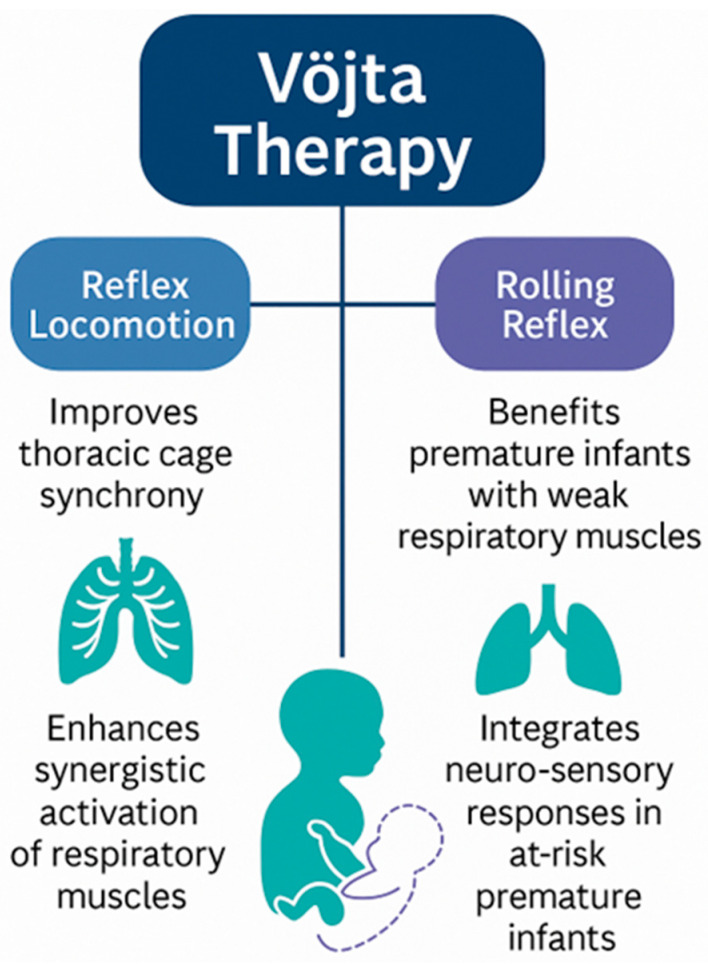
Vöjta-based interventions and their physiological effects.

Reflex-based approaches, particularly those inspired by Vöjta therapy, aim to activate innate neuromotor patterns that facilitate respiratory muscle engagement. By stimulating specific trigger zones, these methods seek to influence not only the respiratory system but also global motor organization and autonomic regulation. One of the most relevant contributions in this area comes from a Spanish research team that applied Vöjta reflex rolling to preterm infants with RDS or BPD. Their work showed improvements in oxygenation, reduction in respiratory distress signs and enhanced neuromotor organization shortly after therapy sessions [10]. These effects suggest that reflex-based interventions may support respiratory mechanics through improved intercostal activation and diaphragmatic recruitment (Figure 2).

Additional insight comes from a randomized trial combining lung squeezing with reflex rolling, where the intervention produced greater increases in SpO_2_ than lung squeezing alone [14]. This suggests a potential synergistic effect when respiratory techniques are integrated with reflex-based neuromotor activation.

Although the evidence base remains smaller than for manual or postural techniques, the available data indicate that reflex-based interventions may be particularly beneficial in infants with altered thoracoabdominal synchrony, weak respiratory muscle recruitment or delayed neuromotor development.

## 6. Multimodal Rehabilitation Strategies Integrating Respiratory, Postural and Motor Components

Some of the most promising interventions identified in this review are those that combine respiratory physiotherapy with broader developmental and postural strategies. These multimodal approaches aim to address the complex physiological and neuromotor needs of premature infants more comprehensively than isolated methods.

A particularly notable example is a structured early rehabilitation program implemented in a neonatal unit in China, which included respiratory physiotherapy, neuromotor stimulation, postural management and orofacial therapy. Infants receiving this combined intervention showed significantly shorter hospital stays, reduced oxygen supplementation and fewer days of mechanical ventilation. The program also yielded a lower incidence of BPD, making it one of the most impactful interventions identified [13].

Another relevant contribution originates from a Brazilian study exploring the combination of chest physiotherapy with motor stimulation activities. This integrated approach produced stronger improvements in cardiovascular and respiratory stability than respiratory techniques alone, suggesting that the synergy between motor control and respiratory mechanics can enhance physiological regulation [9].

**Figure 3:**
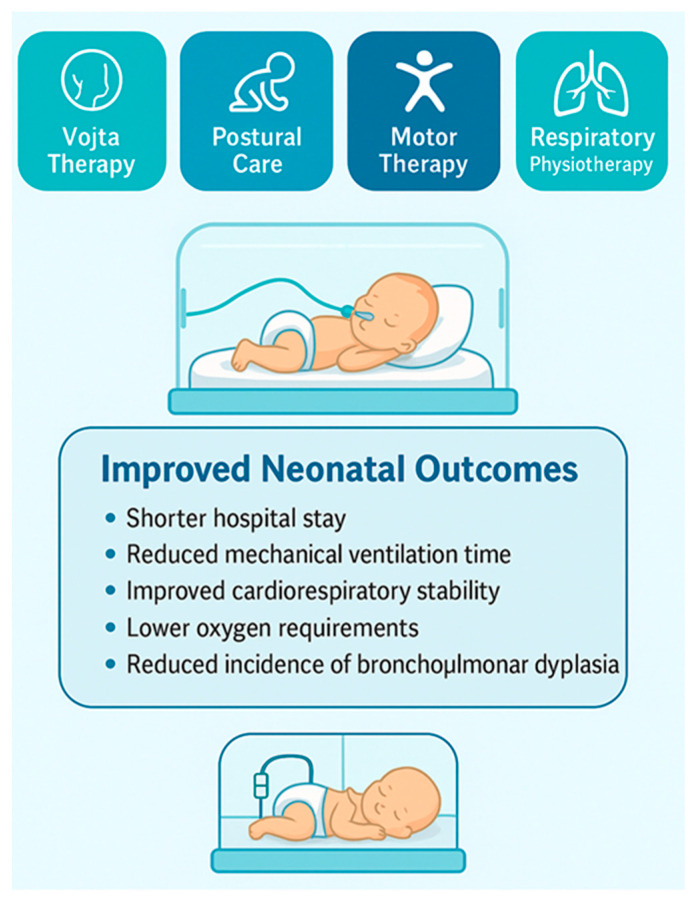
Synergistic effects of multimodal rehabilitation in preterm infants.

Such evidence highlights the potential of multimodal strategies to improve clinical outcomes by addressing not only the respiratory system but also neuromotor maturation, behavioral state organization and overall cardiorespiratory stability (Figure 3).

## 7. Synthesis of Evidence Across Respiratory Physiotherapy Modalities

When integrating findings across all included trials, several consistent patterns emerge regarding the effects of respiratory physiotherapy in preterm infants:

### 7.1. Immediate Physiological Benefits

- Increases in SpO_2_ [14,15,16,17,18,19,20,22].
- Reductions in RR and improved heart rate stability [16,17,18].
- Better breath sounds and improved auscultatory findings [14,20].
- Improved respiratory support parameters
- Faster radiological resolution of atelectasis [14,20].
- Decreased FiO_2_ requirements [19,22].
- Enhanced ventilatory patterns in ventilated preterm infants [15,17].

### 7.2. Medium- to Long-Term Clinical Improvements in Multimodal Programs

- Decreased hospitalization duration [10,13,23].
- Reduced mechanical ventilation days [13,23].
- Lower incidence of BPD in one comprehensive intervention trial [10].

### 7.3. Safety

All modern studies reported excellent tolerance and no significant adverse events, reinforcing that physiotherapy techniques—when performed appropriately—are safe for preterm infants. Historical concerns stem from outdated techniques no longer representative of current practice [11].

## 8. Discussion

The purpose of this comprehensive review was to integrate current evidence on respiratory physiotherapy in preterm infants with RDS or BPD, clarifying its physiological effects, clinical relevance and safety. Overall, the findings suggest that respiratory physiotherapy, whether through manual expiratory modulation, postural strategies or neurodevelopmentally oriented techniques, can produce meaningful short-term improvements in gas exchange, ventilation efficiency, secretion clearance and physiological stability. Although variability between trials prevents firm conclusions regarding superiority of one technique over another, the consistency of observed benefits reinforces its potential role as an adjunctive therapy within neonatal respiratory care.

The observations of this review align with findings from earlier systematic and narrative reviews on neonatal respiratory physiotherapy. Over the past decade, only three systematic reviews have addressed this topic in depth, highlighting both the scarcity of high-quality evidence and the persistent heterogeneity of methodologies. A recent synthesis, published in 2023 [6], included a mix of randomized clinical trials, quasi-experimental studies and case–control designs, reporting improvements in oxygenation and respiratory effort following physiotherapy interventions. Another sytematic review, focused largely on postural and airway-clearance approaches, incorporated randomized and crossover trials and concluded that physiotherapy may reduce respiratory workload and facilitate ventilation in premature infants [24]. These trends are consistent with the findings of this comprehensive review.

The methodological approaches of previous reviews also anticipated some of the challenges encountered here. Earlier syntheses relied on broad tools to appraise study quality [6,25], highlighting risks of bias and variability in intervention delivery. The present review confirms these limitations: although the evidence supports potential clinical benefits, the methodological heterogeneity of primary studies continues to impede more definitive recommendations.

Across the literature, respiratory and motor physiotherapy techniques have demonstrated beneficial effects on cardiorespiratory stability in preterm neonates. Research integrating thoracic and motor stimulation showed improvements in cardiovascular parameters and secretion clearance in preterm infants with RDS, supporting the physiological rationale for combining respiratory and motor components in neonatal care [9]. Similarly, a case report examining thoracic compressions and the EFIT described notable improvements in bronchial secretion clearance in infants with acute respiratory deterioration [21], which aligns with trends observed in several randomized trials.

Conventional respiratory physiotherapy, autogenic drainage and prolonged slow expiration have also been shown to assist in reducing ventilatory support requirements and reversing atelectasis. These effects are coherent with clinical improvements documented in studies employing positive expiratory pressure devices, where enhancements in respiratory parameters and gradual reductions in oxygen dependence were observed throughout the course of hospitalization [17,26].

Nevertheless, not all physiological markers responded uniformly across studies. For example, while some investigations documented improvements in the fraction of FiO_2_ after physiotherapy, others reported no significant changes, likely reflecting differences in therapeutic intensity, duration or underlying lung pathology rather than contradictory evidence. Pain and discomfort also appeared unaffected by gentle, developmentally respectful techniques, reinforcing their suitability for fragile preterm infants [11].

Although manual respiratory physiotherapy techniques demonstrated overall safety and beneficial effects, their mechanisms of action and clinical applicability differ. Techniques such as PLE may be better tolerated in very immature infants due to lower intrathoracic pressure fluctuations, whereas EFIT requires greater technical precision, particularly under positive airway pressure. Lung squeezing and mobilization techniques may provide rapid mechanical effects but should be applied cautiously in extremely preterm infants with unstable thoracic compliance.

Postural interventions likewise demonstrated promising effects. A study investigating various body positions in preterm infants receiving pressure CPAP reported improvements in oxygenation and ventilation distribution [12], supporting the inclusion of postural optimization within multimodal rehabilitation strategies. The consistency between postural and manual approaches highlights the interdependence of thoracic mechanics, diaphragmatic function and global respiratory efficiency in premature newborns.

Clinical response to respiratory physiotherapy is likely to vary according to gestational age, severity of respiratory disease and type of respiratory support. For example, the effects of prone positioning may differ between mechanically ventilated infants and clinically stable neonates receiving CPAP [22], while manual techniques may interact differently with varying levels of positive end-expiratory pressure in ventilated patients [16,17,18,20]. Similarly, reflex-based interventions may be better tolerated in later preterm infants than in extremely preterm neonates with unstable thoracic wall mechanics, as suggested by studies including more clinically stable populations [13,23]. These considerations highlight that respiratory physiotherapy techniques should not be interpreted as interchangeable across patient groups and underscore the importance of individualized clinical application.

Most studies assessing manual and postural respiratory physiotherapy techniques focused on immediate physiological responses, such as oxygenation and respiratory rate [14,15,16,17,18,19,20,22]. Evidence regarding the persistence of these effects and their impact on longer-term clinical outcomes—such as duration of ventilation, recurrence of atelectasis or development of bronchopulmonary dysplasia—remains limited for these approaches. In contrast, multimodal rehabilitation programs more frequently reported medium-term outcomes, including ventilation duration and length of hospital stay [13,21,23], which may partly explain the more consistent sustained benefits observed with these interventions.

Although formal statistical analysis was beyond the scope of this review, interpretation of the findings should consider the sample size of the included studies. Most trials involved relatively small to moderate sample sizes, which substantially limits statistical power. Evidence suggests that studies with limited sample sizes are primarily able to detect large treatment effects, while smaller or moderate effects—despite potential clinical relevance—may remain undetected [27]. Consequently, the absence of statistically significant differences in some outcomes should not be interpreted as evidence of ineffectiveness, but rather as a reflection of limited power within the available evidence base.

Most of the available evidence supporting manual and postural respiratory physiotherapy techniques relates to acute or subacute phases of lung disease, particularly RDS or post-extubation contexts. Evidence specific to infants with established BPD is more limited and heterogeneous, and conclusions regarding technique-specific effectiveness in this population should therefore be drawn with caution.

It is important to distinguish between RDS and BPD when interpreting the effects of respiratory physiotherapy. RDS is primarily characterized by surfactant deficiency, alveolar instability and atelectasis, where the main therapeutic goals are to improve lung aeration and gas exchange. In contrast, established BPD is associated with chronic inflammation, altered lung growth, increased chest wall stiffness and restrictive mechanics, which may influence both tolerance to chest manipulation and the expected response to physiotherapy interventions.

Safety remains a central concern in the application of respiratory physiotherapy in preterm neonates. Although historical cases described iatrogenic rib fractures following inappropriate chest physiotherapy techniques [11], no such adverse events were reported in recent trials employing gentle, synchronized, developmentally guided interventions. This shift reflects an evolution in neonatal physiotherapy practice, prioritizing controlled expiratory flow modulation and biomechanically safe handling. The absence of increased intracranial hemorrhage risk in infants treated with early combined rehabilitation further supports the safety of physiotherapy in this population [10].

Although several studies reported short-term physiological improvements following respiratory physiotherapy, neutral or minimal effects were also observed in a number of trials. In some cases, no significant changes were detected in oxygenation parameters, respiratory effort or radiological resolution of atelectasis, suggesting that therapeutic effects may be modest or highly context-dependent. Moreover, infants with more advanced or established bronchopulmonary dysplasia frequently showed limited or absent response to isolated manual or postural techniques.

In addition, while respiratory physiotherapy was generally reported as safe, potential risks should be considered, particularly in extremely preterm or clinically unstable infants. These include overstimulation, transient alterations in intrathoracic pressures and possible haemodynamic effects, underscoring the need for careful technique selection, clinical expertise and continuous monitoring. Taken together, these findings highlight the importance of a cautious and individualized approach, and suggest that the absence of measurable benefit in some contexts should not be overlooked.

### 8.1. Limitations

Several limitations must be acknowledged.

First, although the search strategy was structured and conducted across major biomedical databases, exhaustive systematic retrieval was not the objective of this article type; therefore, some relevant studies may not have been captured.

Second, the available evidence remains constrained by small sample sizes, predominantly single-center designs and short follow-up periods, which limit generalizability and prevent conclusions regarding long-term respiratory or neurodevelopmental outcomes.

Third, substantial heterogeneity exists across studies in intervention protocols, treatment duration, outcome measures, postnatal age, ventilation status and definitions of bronchopulmonary dysplasia, reflecting variability in neonatal clinical practice and reporting and precluding quantitative synthesis.

Fourth, study selection and data extraction were not performed independently by two reviewers, which may represent a methodological limitation inherent to the narrative design of this review.

Fifth, the absence of a formal risk-of-bias assessment represents a limitation of this review. As a result, interpretation relies on qualitative appraisal of methodological features and consistency of findings rather than hierarchical evidence grading.

Sixth, the heterogeneity of patient populations across studies—including differences in gestational age, severity of respiratory failure and type of respiratory support—limits the generalizability of the findings and precludes subgroup-specific conclusions. In this context, the effectiveness and applicability of manual respiratory physiotherapy techniques may vary according to clinical profile, thoracic wall stability and respiratory support settings, with some techniques being less suitable in extremely preterm or clinically unstable infants.

Finally, interpretation of the available evidence is further limited by the substantial lack of standardization in intervention protocols, including variability in technique application, session duration and frequency, which restricts comparability across studies.

### 8.2. Clinical Implications

Clinical application of respiratory physiotherapy in preterm infants should be individualized and context-dependent. Available evidence suggests that these interventions may be most relevant during acute or subacute phases of respiratory disease, such as respiratory distress syndrome or the post-extubation period, where the primary goals include improving lung aeration, secretion clearance and ventilation homogeneity.

Infants with airway obstruction, position-dependent atelectasis or transient ventilatory instability may derive greater short-term benefit from selected manual or postural techniques, whereas responses in infants with established or advanced bronchopulmonary dysplasia appear more variable and should be interpreted with caution. Technique selection should take into account gestational age, thoracic wall stability, disease severity and type of respiratory support, particularly in the presence of positive airway pressure.

Given the vulnerability of this population, respiratory physiotherapy should be delivered by professionals with specific training in neonatal care, advanced knowledge of respiratory mechanics and the ability to continuously monitor physiological responses. Appropriate expertise is essential to minimize potential risks, adapt interventions in real time and ensure safe, goal-directed application.

## 9. Conclusions

Respiratory physiotherapy emerges as a valuable adjunct within the multidisciplinary management of preterm infants with respiratory disease, particularly in acute conditions such as respiratory distress syndrome. In contrast, evidence regarding its effectiveness in infants with established bronchopulmonary dysplasia remains more limited and heterogeneous, reflecting differences in underlying pathophysiology and therapeutic goals.

The techniques described in the literature consistently demonstrate short-term improvements in oxygenation, ventilation distribution, secretion clearance and overall physiological stability. When delivered with appropriate clinical expertise, these interventions appear safe even in extremely preterm and medically fragile infants.

Despite encouraging evidence, the field remains characterized by methodological heterogeneity, variability in treatment protocols and limited sample sizes. These factors constrain definitive conclusions regarding the optimal techniques, timing and dosing of physiotherapy interventions.

Overall, respiratory physiotherapy represents a promising, non-pharmacological and non-invasive strategy that can complement respiratory support and medical and nursing care, help reduce complications and potentially contribute to improved early respiratory outcomes in preterm infants.

## 10. Future Directions

Future research on respiratory physiotherapy in preterm infants must prioritize methodological rigor, standardized intervention protocols and long-term outcome assessment. High-quality randomized clinical trials, with adequately powered sample sizes, are needed to determine the relative effectiveness of specific techniques, establish optimal treatment frequency and dosing and clarify which physiological subgroups derive the greatest benefit. Harmonizing definitions, intervention components and outcome measures across studies would facilitate comparability and allow for more robust meta-analytic integration.

A deeper understanding of the mechanistic effects of respiratory physiotherapy is also required. Advanced imaging modalities, lung function monitoring and real-time assessments of thoracoabdominal synchrony could help elucidate how manual and postural interventions modulate respiratory biomechanics in preterm infants with immature lung structure.

Longitudinal studies are essential to determine whether early physiotherapy contributes to sustained improvements beyond acute physiological stabilization, including long-term pulmonary function, neuromotor development, hospitalization trajectories and healthcare utilization.

As neonatal care increasingly embraces individualized and family-centered models, evaluating the feasibility of physiotherapy protocols across diverse clinical settings, including low-resource environments, will also be critical.

## Figures and Tables

**Table 1:** Postural Interventions in Preterm Infants: Characteristics, Outcome Measures and Target Effects.

Intervention Characteristics	Specific Measures	Target Effects
Comparison of prone vs. supine positioning in preterm infants on CPAP. Infants remained in each position for 3 h before switching to the alternative posture.	Oxygen saturation, respiratory rate, heart rate. Measurements collected immediately post-intervention and at 30–90 min.	Prone positioning significantly increased oxygen saturation compared with supine, with no significant changes in RR or HR. Improved oxygenation stability.
Assessment of postural effects (supine, prone, lateral positions) on pulmonary function in preterm infants with lung disease.	Pulmonary function tests, including lung compliance and tidal volume, plus heart rate.	Prone and lateral positions improved pulmonary mechanics, reduced work of breathing, and enhanced ventilation distribution.
Evaluation of body positioning (prone, supine, lateral) in preterm infants receiving CPAP.	Oxygen saturation, respiratory pattern, thoracoabdominal synchrony, ventilation distribution.	Prone and lateral positions improved oxygen saturation and thoracoabdominal synchrony, providing more homogeneous ventilation distribution.

CPAP: Continuous positive airway pressure.

## Data Availability

No new data were created.

## Supplementary Materials

The following supporting information can be downloaded at: https://www.mdpi.com/article/10.3390/jcm15010343/s1, Table S1: Characteristics and outcomes of studies included in the comprehensive review.
