# Supplementary material for: Respiratory Physiotherapy in Preterm Neonates with Bronchopulmonary Dysplasia or Respiratory Distress Syndrome: A Comprehensive Review of Clinical Evidence and Therapeutic Implications
Source: J Clin Med. 2026 Jan 2;15(1):343. doi: 10.3390/jcm15010343 (PMC12786764; doi:10.3390/jcm15010343)
Supplement: Supplementary file 1 [file jcm-15-00343-s001.zip › jcm-4046807-supplementary.pdf]

Table S1. Characteristics and outcomes of studies included in the comprehensive review

Author	Year	Sample characteristics	Intervention	Comparator	Outcome measures	Main findings / Conclusions
Kole & Metgud	2014	n=60 preterm neonates; three groups (n=20 each)	Conventional respiratory physiotherapy; lung compression; Vöjta reflex rolling	Comparison between techniques	SpO_2, PaO_2, SaO_2, chest X-ray	All techniques were safe and improved oxygenation; no significant differences between groups.
Mohamed et al.	2014	n=60 preterm neonates with RDS on mechanical ventilation	Respiratory physiotherapy + medical care	Medical care alone	HR, RR, BP, PaO_2, $PaCO_2$, pH, chest X-ray	Physiotherapy group showed greater improvement in cardiorespiratory parameters without adverse effects.
Neha & Neha	2014	n=30 mechanically ventilated preterm neonates with atelectasis	Lung compression technique	Conventional chest physiotherapy + suction	Oxygenation, respiratory pattern, X-ray	Lung compression was more effective and less stressful than conventional physiotherapy.
Mohamed et al.	2015	n=60 preterm neonates with RDS on mechanical ventilation	Respiratory physiotherapy + medical care	Medical care alone	HR, RR, BP, blood gases, imaging	Significant improvement in physiological variables in the physiotherapy group; no adverse events.
Gómez-Conesa et al.	2015	n=45 preterm neonates with RDS	Vöjta therapy + standard NICU care	Standard NICU care	Ventilation days, hospital stay, PERI score	Vöjta therapy reduced ventilation duration, hospitalization and perinatal risk.

Author	Year	Sample characteristics	Intervention	Comparator	Outcome measures	Main findings / Conclusions
Mehta et al.	2016	n=60 preterm neonates with RDS (ventilated and extubated)	Postural drainage, percussion, vibration, suction	Baseline condition	HR, RR, SpO_2, PaO_2/FiO_2, $AaPO_2$, X-ray	Physiotherapy improved respiratory parameters; suction caused transient instability.
Utario et al.	2017	n=15 preterm neonates on CPAP	Prone positioning	Supine positioning	SpO_2, HR, RR	Prone position significantly improved oxygen saturation.
Liu et al.	2021	n=51 preterm neonates	Multimodal early rehabilitation program	Routine care	Oxygen therapy duration, complications, hospital stay, neurodevelopment	Combined rehabilitation improved short-term clinical outcomes.
Tana et al.	2023	n=133 preterm neonates <30 weeks GA, non-intubated	Individualized respiratory facilitation and PSE	Postural program alone	SpO_2, FiO_2, ventilation support, hospital stay	Respiratory physiotherapy was safe and reduced respiratory support trends.

Author	Year	Sample characteristics	Intervention	Comparator	Outcome measures	Main findings / Conclusions
de Macedo et al.	2024	n=43 preterm neonates	EFIT technique	Conventional respiratory physiotherapy	SpO_2, HR, RR, VT, pain, ventilatory parameters	EFIT reduced RR and pain and improved oxygenation compared with conventional therapy.

($AaPO_2$, alveolar–arterial oxygen gradient; BP, blood pressure; CPAP, continuous positive airway pressure; EFIT, expiratory flow increase technique; FiO_2, fraction of inspired oxygen; GA, gestational age; HR, heart rate; NICU, neonatal intensive care unit; $PaCO_2$, arterial carbon dioxide pressure; PaO_2, arterial oxygen pressure; PERI score, Perinatal Risk Inventory score; PSE, prolonged slow expiration; RR, respiratory rate; RDS, respiratory distress syndrome; SaO_2, arterial oxygen saturation; SpO_2, peripheral oxygen saturation; VT, tidal volume)
